# Differentiation of *Amaranthus* Species and Estimation of Their Polyphenolic Compounds and Antioxidant Potential Using Near-Infrared Spectroscopy

**DOI:** 10.3390/plants13233370

**Published:** 2024-11-30

**Authors:** Svetoslava Terzieva, Neli Grozeva, Milena Tzanova, Petya Veleva, Mariya Gerdzhikova, Stefka Atanassova

**Affiliations:** Faculty of Agriculture, Students’ Campus, Trakia University, 6000 Stara Zagora, Bulgaria; n.grozeva@trakia-uni.bg (N.G.); milena.tsanova@trakia-uni.bg (M.T.); petya.veleva@trakia-uni.bg (P.V.); mariya.gerdzhikova@trakia-uni.bg (M.G.)

**Keywords:** *Amaranthus albus* L., *Amaranthus blitum* L., *Amaranthus deflexus* L., *Amaranthus hybridus* L., *Amaranthus retroflexus* L., NIR spectroscopy, SIMCA

## Abstract

*Amaranthus* species are rich in protein, fiber, minerals, and other nutrients and have various health benefits. The genus is taxonomically difficult due to the high phenotypic plasticity and the spontaneous interspecies introgression and hybridization between species. The purpose of this study is to evaluate the possibilities of near-infrared spectroscopy (NIRS) for the taxonomic differentiation of some of the species common in Bulgaria and estimate their polyphenolic compounds. Tested samples were collected from six Bulgarian floristic regions: *Amaranthus albus* L., *A. blitum* L., *A. deflexus* L., *A. hybridus* L., and *A. retroflexus* L. were studied. The NIR spectra of dried and ground leaf and stalk samples were measured by NIRQuest 512 (region 900–1700 nm) using a fiber-optic probe. Soft independent modeling of class analogy (SIMCA) was used to develop the classification models and PLS regression for the quantitative determination of their polyphenolic compounds and antioxidant potential. There were statistically significant differences in the measured values of polyphenolic compounds and antioxidant potential among the tested species. NIRS allowed an accurate determination of these parameters. The performance of developed SIMCA models for the discrimination of species was very high. The precision of determination varied from 98.2 to 100%, and the total accuracy was 98.34%. The results show successful differentiation of the taxonomic species.

## 1. Introduction

Research centered on underexploited foods has increased recently. Amaranth (*Amaranthus* L. spp.) has shown great potential as a source of nutritional and medicinal compounds [1]. They are rich in protein, fiber, minerals, and bioactive compounds such as phenolic acids, polyphenols, unsaturated fatty acids, glucosinolates, soluble peptides, flavonoids, and others [2]. These bioactive substances may possess several health benefits, such as antioxidant, antihyperglycemic, and antihypercholesterolemic effects [3]. The seeds are gluten-free [4]. They are used as a component in various bakery products such as breads, cookies, and savory bites [5,6]. Amaranth leaves and stalks can be consumed in fresh and dry states as an ingredient in sauces, salads, or other dishes [7]. Potions against constipation, anemia, and limb pain are prepared from the roots [8]. The seeds, as well as the other plant organs (leaf, stalk, root), can successfully be part of a healthy human diet.

*Amaranthus* L. is a cosmopolitan genus. Widespread taxa in the genus are characterized by high ecological plasticity, making them an attractive alternative for their cultivation in unfavorable terrains, sometimes displacing conventional crops. In many countries, *Amaranthus* species are cultivated for use as pseudo cereals, vegetables, or ornamentals. Some of them are used as fodder, and others are introduced and naturalized weeds and ruderals. Twelve species are common in Bulgaria [9]. They are found mainly on ruderalized terrains and as weeds in cultivated fields up to 1000 m above sea level. Three of the most common and considered invasive species are *A. albus*, *A. hybridus,* and *A. retroflexus* [10]. The first species grows mainly in areas with vegetable or field crops. It has been cultivated since the end of the 18th century in some parts of Europe. *A. hybridus* is categorized as one of the most economically important weeds in the world [11]. *A. hybridus* and *A. blitum* are both valued as important green leafy vegetables [12]. *Amaranthus retroflexus* is among the most common weeds in Bulgaria together with *A. deflexus* being one of the most widespread ruderals along roads and yards. These two species have good antioxidant potential [13].

The genus is taxonomically difficult due to the high phenotypic plasticity of its species, as well as the spontaneous introgression and hybridization between them. Different robust techniques for the identification of the species have been developed, such as molecular markers and gene expression profiling. These methods are destructive, expensive, labor-intensive, and not applicable for field-level assessment. The discrimination of *Amaranthus* sp. based on the molecular methods of biochemical [14] and DNA analyses has been successfully performed [15].

The development of reliable, rapid, and low-cost methods for the discrimination of different species or the presence of genetically modified crops is very important. In recent years, with the development of near-infrared (NIR) spectroscopy and advanced optical imaging techniques, such as multispectral and hyperspectral imaging in the visible and NIR regions, there has been a rapid increase in the use of these methods in various fields of agriculture. Recent studies related to using NIR spectroscopy for the discrimination of species variety and transgenic species have been published. Several publications by Sohn and co-authors reported the successful application of Vis–NIR spectroscopy and machine learning methods for the discrimination of transgenic *Brassica napus* L. and their hybrids with *B. juncea* [16], discrimination of transgenic canola (*Brassica napus* L.) and their hybrids with *B. rapa* [17], and discrimination of *Brassica napus* varieties [18].

Near-infrared spectroscopy (NIRS) can also be used to assess the chemical composition of plants and seeds. Prediction of the carotenoid content in *Amaranthus* sp. leaf using visible–NIR imaging has been reported [19]. The obtained coefficient of determination was 0.878, and the standard error of estimation was 0.01 μmol/mL. Shruti [20] investigated possibilities for the development of NIR models for estimating oil, proteins, essential amino acids, and fatty acids in the amaranth and buckwheat germplasm. The reported coefficient of determination of the models ranged from 0.72 to 0.929, and the ratio performance deviation value for most of the traits ranged between 2 and 3, indicating good prediction capabilities in the developed model.

The main objective of this study was to evaluate the possibilities of NIR spectroscopy for the taxonomic differentiation of some *Amaranthus* species that are widespread in Bulgaria and the estimation of their polyphenolic compounds and antioxidant potential.

## 2. Materials and Methods

### 2.1. Plant Samples

**Figure 1:**
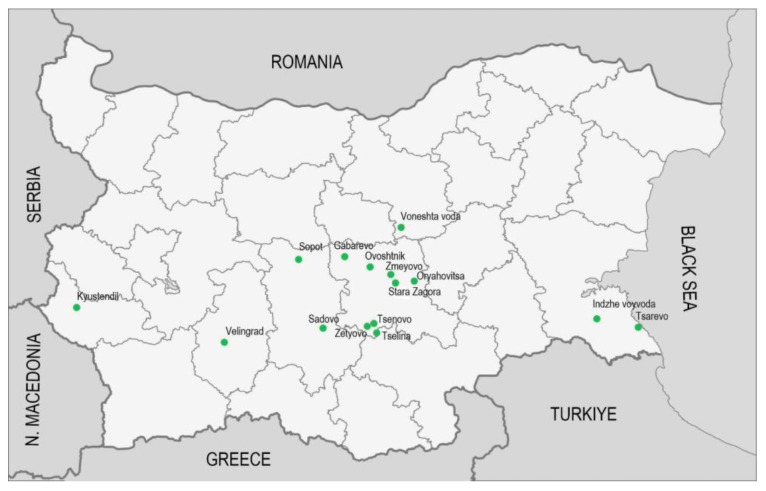
Map of the collection localities.

**Figure 2:**
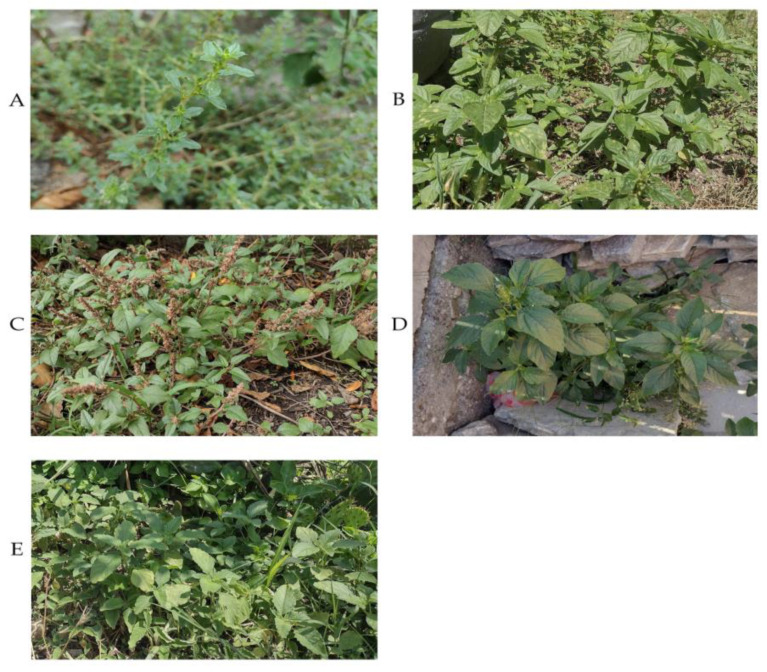
Photographs of the studied species of the genus *Amaranthus* L.: (**A**) *A. albus* L.—Stara Zagora; (**B**) *A. blitum* L.—Kyustendil; (**C**) *A. deflexus* L.—Gabarevo; (**D**) *A. hybridus* L.—Velingrad; (**E**) *A. retroflexus* L.—Voneshta voda.

The tested samples were collected in 2023 from the territory of six Bulgarian floristic regions—Stara Planina (Central), Thracian Lowland, West Frontier Mountains, Strandzha Mountain, Tundzha Hilly Plain, and Rhodope Mountains (West). Five species (*A. albus* L., *A. blitum* L., *A. deflexus* L., *A. hybridus* L., and *A. retroflexus* L.) from 29 local wild-growing populations were studied (Figure 1, Table 1). Figure 2 shows photographs of the studied species of the genus *Amaranthus* L. From each sample, subsamples of leaves and stalks were prepared.

The plant material collected was air-dried in the shade at room temperature. It was ground in a mechanical grinder to a final particle size less than 400 μm. The samples were stored in dark and cool rooms at 16–18 °C prior to chemical and spectral analyses.

### 2.2. Sample Extraction

An amount of each ground sample was weighed on an analytical balance and suspended in 70% ethanol at a ratio of 1:10. The extraction was carried out by ultrasonication for 30 min at 40 °C. After filtration through a 0.45 μm membrane, the solid residue was rinsed with 70% ethanol in triplicate. The alcoholic fractions from each sample were collected and adjusted to a final concentration of 1 mg/mL extract.

The extraction technique of ultrasonication and 70% ethanol as an extracting agent was selected due to the quantity of the extraction of the target compounds [21].

### 2.3. Determination of Radical Scavenging Capacity by DPPH Method

The 2,2-diphenyl-1-picrylhydrazyl radical (DPPH) used was purchased from Sigma-Aldrich (St. Louis, MO, USA). The method described by Tzanova et al. [22] was applied to measure the radical scavenging potential of the alcoholic extracts obtained from different plant parts of the selected species. In brief, 100 μL of the extract was added to 3.9 mL of 100 M solution of DPPH in methanol. The absorption at 517 nm was measured on a Thermo Scientific Evolution 300 spectrophotometer 30 min later. Since the composition of the extracts is complex, the results for their radical scavenging capacity were compared with Trolox and calculated by regression analysis from the linear dependence between the concentration of the Trolox and absorption at 517 nm (R^2^ = 0.9985 for the linearity of the concentration range from 5 to 50 μmol/L). The results were expressed as µmol Trolox equivalent (TE) in 1 g dm of the plant extract.

### 2.4. Determination of Total Phenolic Content (TPC)

The experimental protocol described by Tzanova et al. [22] was followed for the quantification of TPC. In brief, 1 mL of the alcoholic plant extract was mixed with 5.0 mL of Folin–Ciocalteu’s reagent (1:10 dilution). Then, 4 mL of 7.5% Na_2_CO_3_ was added, and the tubes were left at room temperature for 60 min. The absorbance at 765 nm was measured against a blank on a Thermo Scientific Evolution 300 spectrophotometer. Gallic acid (Sigma-Aldrich, St. Louis, MO, USA) solutions in 70% ethanol ranging from 10 to 150 μg/mL were used to plot the calibration curve (R^2^ = 0.9991). The TPC of each sample was expressed as milligrams of gallic acid equivalents (GAE) in 1 g of dry matter (dm) of the plant extract.

### 2.5. Determination of Total Flavonoid Content (TFC)

For the quantification of the TFC, the experimental procedure described by Dinev et al. [23] was followed. In brief, 1 mL of the extract, 0.3 mL of 5% NaNO_3,_ and 4 mL of deionized water were mixed. Then, 0.3 mL of 10% AlCl_3_ (after 5 min) and 2 mL of 1 M NaOH (after 6 min) were added in this order. The solution was homogenized and the absorbance was measured against the blank at 510 nm on a Thermo Scientific Evolution 300 spectrophotometer. Standard solutions of catechin hydrate (Sigma Aldrich, St. Louis, MO, USA) in the concentration range from 10 to 150 mg/L were used to plot the calibration curve (R^2^ = 0.9982). The TFC was expressed as mg of catechin equivalent (CE) in 1 g of dm extract.

### 2.6. Determination of Total Condensed Tannin Content (TCT)

The TCT was obtained using vanillin as a reagent and catechin as a standard. The experimental procedure described by Rebeya et al. [24] was followed. In brief, 0.4 mL of the extract was added to 3 mL of 4% methanolic solution of vanillin and 1.5 mL of conc. HCl. The solution was homogenized, and after 15 min, the absorbance was measured against a blank at 500 nm on a Thermo Scientific Evolution 300 spectrophotometer. Standard solutions of catechin hydrate (Sigma Aldrich, St. Louis, MO, USA) in the concentration range from 10 to 150 mg/L were used to plot the calibration curve (R^2^ = 0.9997). The TCT was expressed as mg of catechin equivalent (CE) in 1 g of dm extract.

### 2.7. NIR Measurements and Spectral Data Analysis

NIRS measurements were performed by using a NIR Quest 512 spectrometer (Ocean Optics, Inc., Orlando, FL, USA) in the region of 900–1700 nm, a reflection from a fiber-optics probe. The reflection fiber probe was fixed in the reflection holder to position it perpendicular to the measured surface and at a constant distance to ensure uniform measurement conditions. Three portions of each sample were measured. Several measurements at different parts of the samples were made and averaged to minimize any possible effects of variation in the samples.

Spectral data processing was performed using Pirouette 4.5 software (Infometrix, Inc., Bothell, WA, USA). The soft independent modeling of class analogy (SIMCA) was used as the classification method. The class variable was assigned to each analyzed sample according to the respective *Amaranthus* species. The SIMCA model applied was based on principal components analysis (PCA). The number of significant components of each class was evaluated using leave-one-out cross-validation. The probability threshold was set to 0.95. The probability threshold is a value used to determine whether a sample belongs to a certain class or not. The precision, sensitivity (recall), and total accuracy were used as the classification model performance indicators. Precision measures the ability of the model to identify samples from a particular class. It is calculated as the number of correct predicted samples from the respective class divided by the total number of samples of that class. Recall is the number of correct predicted samples from a particular class divided by the sum of correct predicted plus samples from other classes, predicted as belonging to that class. Total accuracy measures the number of correct predictions divided by the number of predictions made. These indicators were multiplied by 100 to become a percentage.

PLS regression was used to develop equations for polyphenolic compounds and antioxidant potential determination. The calibration equations for each parameter were developed and validated with leave-one-out cross-validation. The leave-one-out method is recommended when a few samples are used to build the calibration equations. One sample from the calibration dataset was left out and an equation was developed with the remaining samples. This equation was used to predict the tested parameter for the omitted sample. This procedure was repeated until each sample was used once as a validation sample, and the correlation coefficient between predicted and reference values and standard error of cross-validation SECV was calculated. The prediction capacity of each calibration equation was evaluated using statistical parameters from the calibration procedure: R—multiple correlation coefficients between reference values and NIR predicted values; SEC—standard error of calibration; and SECV—standard errors of cross-validation. 

## 3. Results and Discussion

### 3.1. Differentiation of Species by Near-Infrared Spectra

**Figure 3:**
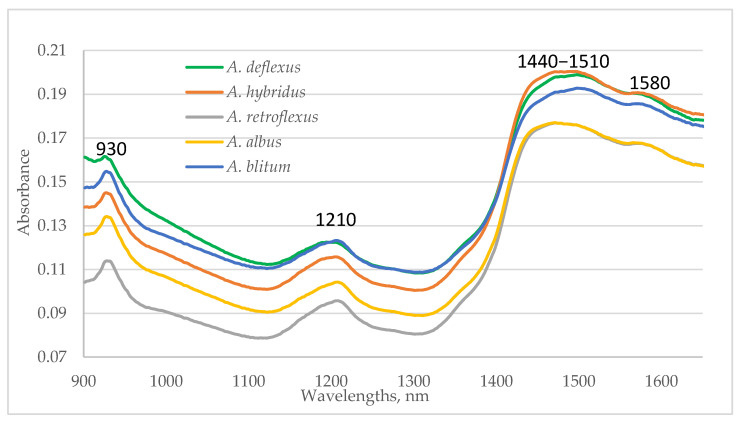
Average spectra of investigated *Amaranthus* species.

The average spectra collected from the leaves and stalks of five investigated *Amaranthus* species are shown in Figure 3. Since the studied samples are from the same botanical genus, their spectral characteristics are similar. The absorption maxima were observed around 930, 1210, 1430–1510, and 1580 nm. The biggest maxima in the region around 1440 nm might be assigned to the O–H vibration from the water and fiber fraction of the plant. Differences in spectra at 1450–1510 nm could be related to N-H bands of protein constituents. The absorption at wavelength regions around 930 and 1210 nm could be related to the C–H vibrations and at 1580 nm to the O-H vibrations [25].

Plants were collected in different stages of vegetation, from different floristic regions and ecological conditions, and places with different altitudes. We can consider that the analyzed samples are typical for the Bulgarian population of the studied species. The differences between the spectra of the tested studied species are due mainly to the differences in their chemical composition. For example, the crude fiber contents of the samples from *A. retroflexus* L. and *A. albus* L. were lower than those of *A. deflexus* L. and *A. hybridus* L. The protein content was the highest in samples from *A. blitum* L. Therefore, the NIR spectra contain information specific to the studied *Amaranthus* species.

The results of the SIMCA models for the discrimination of *Amaranthus* species are presented in Table 2. The performance of the developed SIMCA models was very high. The precision varied from 88.46 to 100%, the recall varied from 90 to 100%, and the total accuracy was 98.34%. The results show successful differentiation of the plant materials by taxonomic species. Therefore, the NIR spectra contain sufficient information for the identification of the studied species of the genus *Amaranthus*. NIR spectroscopy, in combination with chemometric methods for discrimination could be used to quickly classify species for monitoring their potential applications.

This study confirmed the results of Sohn et al. [26] that the discrimination and classification of five *Amaranthus* species using portable VIS–NIR spectroscopy are possible in combination with chemometrics techniques. The authors reported a classification accuracy between 71% and 99.7% after the cross-validation.

These results are important for plant taxonomy, as well as for agriculture. Some Amaranthus species are common weeds in corn, sunflower, and other crops. Determining their taxon is important for controlling weeds with the most appropriate herbicide for certain weeds. Spectral methods for plant differentiation could have good applications in precision agriculture, especially when using portable handheld spectrometers and drone cameras.

### 3.2. Polyphenolic Compounds and Antioxidant Potential of Tested Amaranthus Species

The mean values and standard deviation of each measured parameter of tested *Amaranthus* species are reported in Table 3. There were statistically significant differences in the measured values among tested species. The highest value for radical scavenging capacity was observed for *A. hybridus* leaves and stalks, while the lowest was for *A. lividus* leaves and stalks. The radical scavenging capacity of leaves was higher than that of stalks. The total phenolic content, total flavonoid content, and total condensed tannins were much higher for leaves than the content in stalks for all tested species. The highest value of the total phenolic content was measured for *A. hybridus* leaves and stalks. Total flavonoid content values were observed in *A. retroflexus* leaves and total condensed tannins in *A. albus* leaves, respectively. 

The results follow the findings of Nana et al. [27], Li et al. [28], and Bang et al. [29]. Differences in the values of measured radical scavenging activities, total tannin contents, total flavonoids, and total polyphenols between *A. hybridus* and *A. cruentus* were found [27]. The *A. hybridus* extract showed the best antioxidant activities. Li et al. [28] investigated the phytochemical profiles and antioxidant activities of different parts, including the leaves, stalks, seeds, flowers, and sprouts of three *Amaranthus* species (*A. hypochondriacus*, *A. caudatus*, and *A. cruentus*). They reported the differences in the values of the measured parameters between the leaves and stalks, as well as between species. Bang et al. [29] investigated the phenolic content of nine *Amaranthus* species: *A. hypochondriacus*, *A. cruentus*, *A. caudatus*, *A. tricolor*, *A. dubius*, *A. blitum*, *A. crispus*, *A. hybridus*, and *A. viridis*. They reported significant variability between the genotype variables for 17 types of polyphenols.

### 3.3. NIR Spectroscopy Estimation of Polyphenolic Compounds and Antioxidant Potential

**Figure 4:**
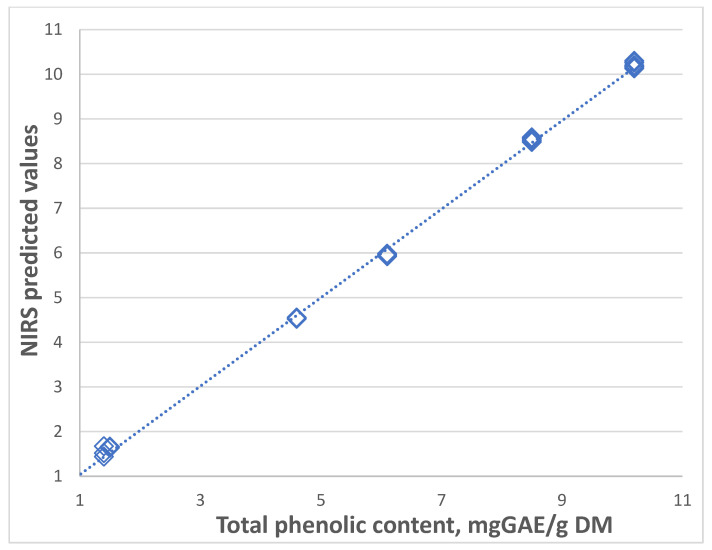
NIR spectroscopy prediction of total phenolic content.

Table 4 shows the statistics for the NIRS equations for the prediction of polyphenolic compounds and the antioxidant potential of the tested species. The results of the NIRS analysis were comparable with the reference chemical methods. The determination accuracy was very high. For each of the tested parameters, the correlation coefficient Rcv was 0.99, and the determination errors were small. A graphical illustration of the accuracy of the NIR spectroscopy prediction of the total phenolic content is presented in Figure 4. 

Polyphenolic compounds contain a large number of hydrogenous bonds, which have absorption bands in the NIR region. In particular, the first overtone of the O-H bonds is in the area of 1300–1600 nm, where the largest absorption of the tested samples is obtained. This would explain the very good accuracy of the determination of polyphenolic compounds in the studied plants.

NIR spectroscopy could be used as an alternative to traditional analytical methods for the determination of bioactive compounds directly in the plant matrix without complicated sample preparation and using toxic reagents. NIRS analysis would allow the rapid screening of plants according to their antioxidant activity and polyphenolic composition for food or medicinal purposes. This would also have important applications in breeding programs for selecting plants with desirable qualities.

In the future, recently developed portable and inexpensive NIR instruments will allow for the fast evaluation of polyphenolic content and antioxidant potential of plants not only in dry samples but also directly in the field.

## 4. Conclusions

The spectral characteristics of the studied *Amaranthus* species are similar, but at the same time, differences are observed among the spectra of the different species. These differences allowed for a successful differentiation of the plant materials by taxonomic species based on the NIR spectra and chemometric classification methods.

There were statistically significant differences in the measured values of the polyphenolic compounds and antioxidant potential among the tested *Amaranthus* species. NIR spectroscopy allowed for a very accurate determination of these parameters.

The results of this study demonstrated that NIR spectroscopy applied with chemometric methods could be used as a green technology for the identification and quality assessment of the investigated species.

## Figures and Tables

**Table 1:** Coordinates of the studied localities of *A. albus* L., *A. blitum* L., *A. deflexus* L., *A. hybridus* L., and *A. retroflexus* L.

No.	Location	Floristic Regions	Coordinates	Altitude (m)	Herbarium Voucher
***Amaranthus albus* L.**					
1	Gabarevo	Stara planina (Central)	42°37′59.936″ N 25°9′28.000″ E	465	SOA 063562
2	Sadovo	Thracian Lowland	42°07′31.488″ N 24°56′04.471″ E	158	SOA 063561
3	Sopot	Thracian Lowland	42°39′18.410″ N 24°44′41.317″ E	521	SOA 063560
4	Stara Zagora	Thracian Lowland	42°25′54.569″ N 25°37′45.523″ E	220	SOA 063559
5	Tsenovo	Thracian Lowland	42°09′30.128″ N 25°23′43.431″ E	176	SOA 063558
***Amaranthus blitum* L.**					
6	Kyustendil	West Frontier Mountains	42°16′29.173″ N 22°40′49.351″ E	565	SOA 063557
7	Stara Zagora	Thracian Lowland	42°25′53.691″ N 25°38′55.595″ E	209	SOA 063556
***Amaranthus deflexus* L.**					
8	Gabarevo	Stara planina (Central)	42°37′58.474″ N 25°9′25.982″ E	466	SOA 063555
9	Indzhe voyvoda	Strandzha Mountain	42°13′24.685″ N 27°24′58.775″ E	292	SOA 063554
10	Ovoshtnik	Tundzha Hilly Plain	42°34′42.512″ N 25°26′58.566″ E	331	SOA 063553
11	Sopot	Thracian Lowland	42°39′18.410″ N 24°44′41.317″ E	521	SOA 063552
12	Stara Zagora	Thracian Lowland	42°25′53.691″ N 25°38′55.595″ E	209	SOA 063551
13	Tsarevo	Black Sea coast (South)	42°9′44.140″ N 27°51′25.363″ E	25	SOA 063550
14	Tsenovo	Thracian Lowland	42°09′30.128″ N 25°23′43.431″ E	176	SOA 063549
15	Velingrad	Rhodope Mountains (West)	42°1′45.588″ N 23°59′42.383″ E	762	SOA 063548
***Amaranthus hybridus* L.**					
16	Gabarevo	Central Stara planina	42°37′59.936″ N 25°9′28.000″ E	465	SOA 063547
17	Ovoshtnik	Tundzha Hilly Plain	42°34′42.428″ N 25°26′57.549″ E	331	SOA 063546
18	Sadovo	Thracian Lowland	42°07′31.488″ N 24°56′04.471″ E	158	SOA 063545
19	Sopot	Thracian Lowland	42°39′18.410″ N 24°44′41.317″ E	521	SOA 063544
20	Stara Zagora	Thracian Lowland	42°25′55.685″ N 25°38′35.775″ E	218	SOA 063543
21	Velingrad	Rhodope Mountains (West)	42°1′45.588″ N 23°59′42.383″ E	762	SOA 063542
22	Zmeyovo	Thracian Lowland	42°29′41.828″ N 25°36′47.549″ E	414	SOA 063541
***Amaranthus retroflexus* L.**					
23	Indzhe voyvoda	Strandzha Mountain	42°13′24.685″ N 27°24′58.775″ E	292	SOA 063540
24	Oryahovitsa	Thracian Lowland	42°29′8.303″ N 25°48′30.246″ E	234	SOA 063539
25	Sadovo	Thracian Lowland	42°07′31.488″ N 24°56′04.471″ E	158	SOA 063538
26	Sopot	Thracian Lowland	42°39′18.410″ N 24°44′41.317″ E	521	SOA 063537
27	Tselina	Thracian Lowland	42°07′37.258″ N 25°26′39.533″ E	133	SOA 063536
28	Voneshta voda	Stara planina (Central)	42°52′33.488″ N 25°38′27.471″ E	361	SOA 063535
29	Zetyovo	Thracian Lowland	42°08′53.633″ N 25°22′14.986″ E	180	SOA 063534

**Table 2:** Confusion matrix of species discrimination.

	Predicted *A. deflexus*	Predicted *A. hybridus*	Predicted *A. retroflexus*	Predicted *A. albus*	Predicted *A. blitum*	Precision, %	Sensitivity, (Recall)%
***A. deflexus***	69	1	0	0	0	98.57	100
***A. hybridus***	0	55	1	0	0	98.21	96.49
**A. *retroflexus***	0	1	58	0	1	96.67	98.30
***A. albus***	0	0	0	46	0	100	100
***A. blitum***	0	0	0	0	9	100	90

**Table 3:** Polyphenolic compounds and antioxidant potential of tested *Amaranthus* species.

Amaranthus Species	Radical Scavenging Capacity	Total Phenolic Content	Total Flavonoid Content	Total Condensed Tannins
	µmol TE/g dm *	mgGAE/g dm	mgCE/g dm	mgCE/g dm
***A. retroflexus* leaf**	4.0 ± 0.2 ^a^	8.5 ± 0.4 ^a^	**43.0** ± 1.5 ^a^	21.7 ± 0.6 ^a^
***A. retroflexus* stalk**	3.8 ± 0.1 ^ef^	1.4 ± 0.1 ^e^	**29.5** ± 1.1 ^e^	12.1 ± 0.3 ^e^
***A. lividus* leaf**	3.3 ± 0.1 ^ac^	6.1 ± 0.3 ^a b^	29.0 ± 1.0 ^ab^	23.4 ± 0.7 ^ab^
***A. lividus* stalk**	3.3 ± 0.1 ^e^	0.9 ± 0.1 ^ef^	18.2 ± 0.8 ^ef^	13.8 ± 0.4 ^ef^
***A. hybridus* leaf**	**4.8** ± 0.3 ^abc^	**10.2** ± 0.4 ^a bc^	38.4 ± 1.2 ^abc^	35.2 ± 0.8 ^abc^
***A. hybridus* stalk**	**4.5** ±0.2 ^efg^	**1.5** ± 0.1 ^fg^	22.3 ± 0.9 ^efd^	13.2 ± 0.4 ^eg^
***A. albus* leaf**	3.9 ±0.2 ^bcd^	4.6 ± 0.2 ^abcd^	40.9 ± 1.4 ^bd^	**39.5** ± 0.9 ^abcd^
***A. albus* stalk**	3.3 ± 0.1 ^egh^	0.5 ± 0.1 ^efgh^	23.9 ± 0.9 ^efd^	18.0 ± 0.4 ^efg^
***A. deflexus* leaf**	3.8 ± 0.1 ^bd^	**1.5** ± 0.1 ^abcd^	28.1 ± 1.2 ^acd^	24.2 ± 0.7 ^acd^
***A. deflexus* stalk**	3.6± 0.1 ^fgh^	0.2 ±0.1 ^efgh^	23.9 ± 1.0 ^ef^	**18.8** ± 0.5 ^efg^

* Same letter in superscript within the same column represents significant differences at *p* < 0.05. ^a^—between the *A retroflexus* leaf and all other species’ leaf; ^b^—between the *A. lividus* leaf and all other species’ leaf; ^c^—between the *A. hybridus* leaf and all other species’ leaf; ^d^—between the *A. hybridus* leaf and all other species’ leaf; ^e^—between the *A retroflexus* stalk and all other species’ stalk; ^f^—between the *A. lividus* stalk and all other species’ stalk; ^g^—between the *A. hybridus* stalk and all other species’ stalk; ^h^—between the *A. hybridus* stalk and all other species’ stalk.

**Table 4:** Calibration statistics for NIRS estimation of polyphenolic compounds and the antioxidant potential determination using PLS regression.

Parameter	PLS Factors	SECV	Rcv	SEC	Rcal
Radical Scavenging Capacity	2	0.020	0.99	0.018	0.99
Total phenolic content	3	0.115	0.99	0.095	0.99
Total flavonoids content	3	0.081	0.99	0.070	0.99
Total tannins	5	0.137	0.99	0.059	0.99

## Data Availability

All data are available from the corresponding author upon request.
